# Extracorporeal shockwave treatment impedes tooth movement in rats

**DOI:** 10.1186/s13005-018-0181-5

**Published:** 2018-11-12

**Authors:** Phimon Atsawasuwan, Yinghua Chen, Karan Ganjawalla, Albert L. Kelling, Carla A. Evans

**Affiliations:** 1 Department of Orthodontics, University of Illinois at Chicago, College of Dentistry, 801 S. Paulina St, Chicago, Illinois 60612 USA; 2 Harvard School of Dental Medicine, Boston, Massachusetts USA; 3 Raleigh, USA; 4 Department of Orthodontic and Dentofacial Orthopedics, Boston University, Boston, Massachusetts USA

**Keywords:** Tooth movement, ESWT, Shockwave, Orthodontic, Rat

## Abstract

**Background:**

Accelerated tooth movement has been a topic of interest for orthodontic research recently. Surgically facilitated orthodontic treatment has been shown to be an effective approach to accelerate tooth movement; however, it remains invasive, requires additional surgery, and may increase post-operative complications. In this study, we evaluate the effects of extracorporeal shockwave treatment (ESWT), a non-invasive approach to regenerate alveolar bone, on orthodontic tooth movement in rats.

**Materials and methods:**

Seventy-two male rats, aged 10 weeks old, were subjected to 10-cN closed-coil nickel-titanium springs for unilateral maxillary first molar tooth movement. One group of rats received a single treatment of extracorporeal shockwave treatment at 500 impulses at energy flux density 0.1 mJ/mm^2^, with a pulse rate of 5 pulses per second immediately after spring installation while the non-ESWT-treated group served as a control group. The rats were sacrificed at day 3, 7, 14, 21 and 28 for tooth movement evaluation and sample analyses. Faxitron radiography, histological, double bone labeling and gene expression analyses were performed. Serum biochemistry was evaluated at day 3, 7 and 28 of the study. Kruskal-Wallis analysis of variance was used to determine the mean difference among groups, and multiple comparisons were analyzed by Mann-Whitney-U tests with a significance level = 0.05.

**Results:**

The results demonstrated that tooth movement in the ESWT-treated rats (0.11 ± 0.07 mm) was impeded compared to the tooth movement in the non-ESWT-treated rats (0.44 ± 0.09 mm). ESWT up-regulated several osteoblastic and osteoclastic gene markers and cytokines; however, the effects on osteoclasts were only transient. Double-fluorescence bone labeling demonstrated that osteoblastic activity increased after ESWT treatment. There was no difference in systemic RANKL/OPG ratio between groups.

**Conclusions:**

ESWT at 500 impulse at energy flux density 0.1 mJ/mm^2^ increased osteoblast and osteoclast activities and imbalanced bone remodeling resulting in impeded tooth movement in rats.

## Background

Several approaches such as surgery, vibration/laser application, administration of biomolecules accelerate tooth movement; however, they demonstrate inconsistent results or provide insufficient evidence to reveal their effectiveness [1–9]. Increased alveolar bone traumatization is often used to maximize physiological healing cascades in a regional acceleratory phenomenon (RAP) with mineral release, localized osteoporosis (osteopenia), and increased osteoclast activities resulting in accelerated tooth movement [9]. However, the definite underlying mechanism and extent of the effect is still controversial [10, 11]. Despite the effectiveness of this approach, it remains invasive, requires additional surgery, and may increase post-operative complications.

Extracorporeal shockwave treatment (ESWT) is a non-invasive modality using a shockwave, an intense and very short energy wave traveling faster than the speed of sound [12]. ESWT has been used safely for augmentation of wound healing of bone and muscle and promotes alveolar bone regeneration, neovascularization, distraction osteogenesis and stem cell differentiation in rats [12–15]. ESWT has been used increasingly in healing of several refractory musculoskeletal disorders [16–18] yet its biologic mechanism is not well understood [12, 13]. Recently ESWT has been reported to induce interleukin-1β (IL-1β), a potent cytokine for bone resorption, during 3 days of tooth movement in rats and no long-term observation was studied [19]. However, a study in human**s** failed to show any accelerated effect on second mandibular mesialization [20] after a single treatment of ESWT. Though ESWT promotes healing and induces tissue regeneration, the effect on tooth movement is controversial with different parameters /applications and tested species and subjects [19, 20]. This study evaluated whether ESWT affects tooth movement in a rat model to gain better insight into underlying mechanisms.

## Methods

### Tooth movement and ESWT application

**Fig. 1:**
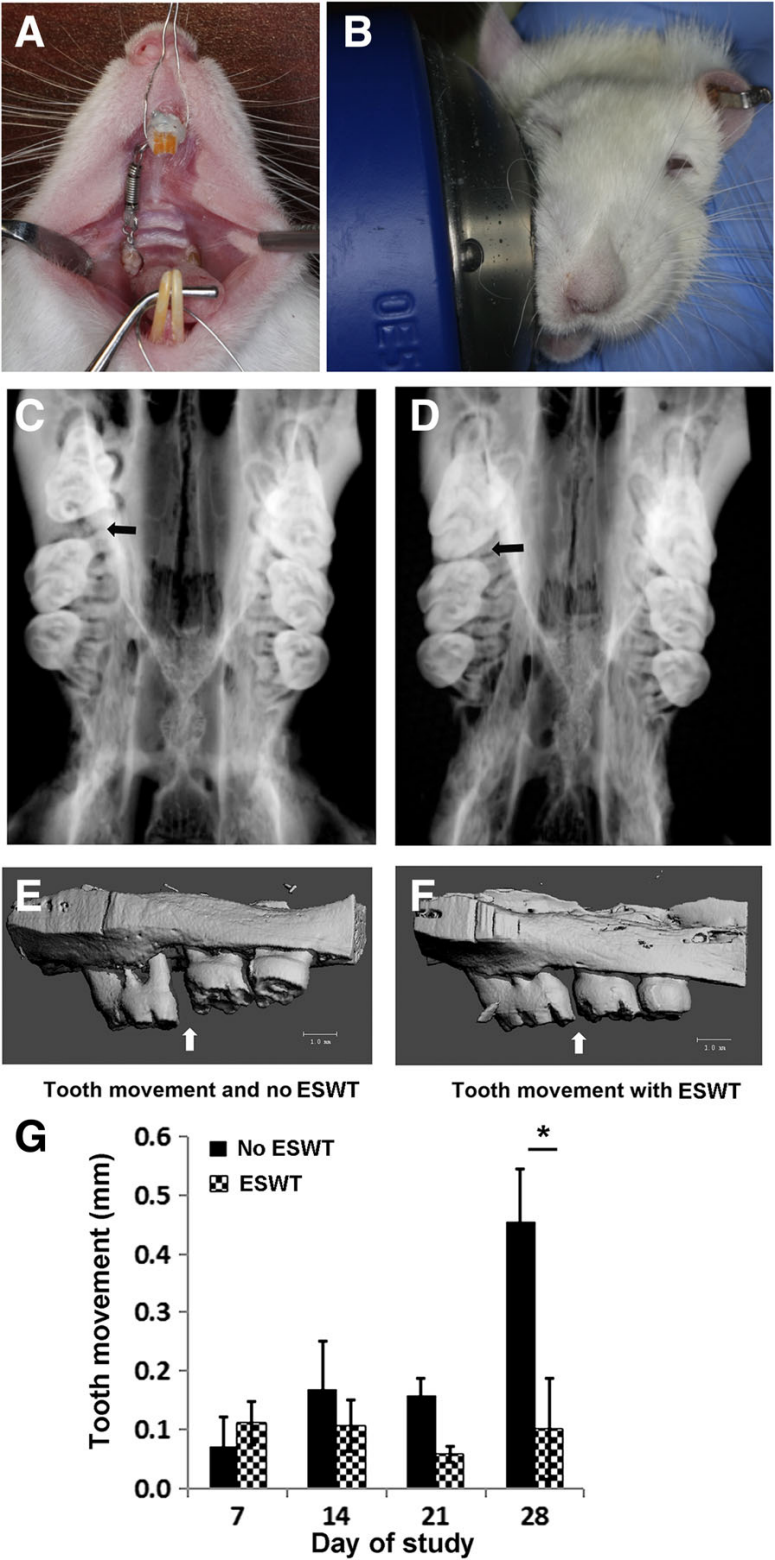
Extracorporeal shockwave treatment and tooth movement. **a** tooth movement in a rat model. **b** Shockwave application on a rat’s maxilla. **c**, **d** Radiographs of rat maxillae showing movement of right first molars at 28 days. **c** A 0.5-mm space (black arrow) detected between first and second upper right molars in a control rat. **d** A 0.08-mm space (black arrow) detected in the corresponding area in a shockwave-treated rat. **e**, **f** Three-dimensional constructed images of rats’ maxillae showing slower tooth movement in a shockwave-treated rat (white arrow, **e**) compared to the non-shockwave control (white arrow, **f**). **g** Distance of tooth movement at different studied timepoints. (ESWT: shockwave therapy, *: *P* < 0.05)

Seventy-two male Sprague-Dawley rats, aged 10 weeks (300-350 g; Charles River Laboratories, Wilmington, MA) were used in this study carried out in accordance with the recommendations in the Guide for the Care and Use of Laboratory Animals of the National Institutes of Health. The protocol was approved by the animal care committee, the University of Illinois at Chicago (ACC 12–183). During the course of the study, the rats were fed ad libitum with powdered rat chow and kept at a 12-h light cycle in the animal facility of the University of Illinois at Chicago. After anesthetized with ketamine and xylazine, all rats were subjected to 10-cN closed-coil springs (American Orthodontics, Sheboygan, WI) to mesialize their right maxillary first molars (ortho) [21] while the left maxillary first molars were left untouched (Fig. 1a) and served as the no orthodontic force (no ortho) control. Stainless steel ligature was used to ligate the spring between the first maxillary molar and the maxillary incisors then self-etching primer (3 M Unitek, Maplewood, MN) was applied over the ligations followed by light-cure flowable composite resin (3 M Unitek) to secure the wire and ensure stability. The rats were randomly divided into 2 groups: non-shockwave-treated controls (no ESWT; *n* = 38) and shockwave treated group (ESWT; *n* = 34). In the ESWT group, a single shockwave treatment of focused 500 impulses at energy flux density (EFD) 0.1 mJ/mm^2^, with a pulse rate of 5 pulses per second was applied on the edentulous area on each side of the maxillae (Fig. 1b) [22]. A shockwave device (Vetgold®, Tissue Regeneration Technology LLC, Atlanta, GA) was used to deliver shockwaves on the rats’ maxillae. Double-fluorescence bone labeling by intraperitoneal injection of calcein (10 mg/kg) was performed on day 0 and 24 and with alizarin-red (30 mg/kg) on day 14.

### Faxitron radiography and microcomputed tomogram (μCT) imaging

Faxitron radiographic imaging (Faxitron X-ray Corp, Wheeling, IL) was performed at 3X magnification with 35 kV for 45 s for all samples. Mesial molar movement was determined by comparing the sagittal distance of the molar position on each side relative to the rats’ second molar. The tooth movement measurement was performed with a ruler tool in Adobe photoshop program (Adobe Systems, San Jose, CA). Hemimaxillae were then scanned with Scanco μCT40 (Scanco Medical, Switzerland) at a parameter of 70 kV, 114 μA, 300 ms and 10 μm voxel size to render 3-dimensional μCT images and bone parameter analysis.

### Gene expression study

At day 7, 14 and 28 of the study, alveolar bone segments mesial to (compression) and distal to (tension) the first maxillary molars were dissected under a stereomicroscope. The corresponding bone samples on the contralateral sides were collected as the no orthodontic force (no ortho) controls. The bone samples were pulverized under liquid nitrogen and kept in TRiZol at − 80° until use. Total RNA extraction was performed with RNeasy kit (Qiagen, Carlsbad, CA), subjected to a nanodrop spectrophotometer to verify the quality of RNA. cDNA was generated with Omniscript RT kit (Qiagen) following the manufacturer’s instructions. To quantify mRNA expression levels, realtime-PCR was performed using sequence-specific Sybergreen primers (Table 1) and normalized using β-actin. The analyses were performed using Quanstudio 3 Real-Time PCR system (Thermo Fisher Scientific, Waltham, MA). Relative expression levels were calculated using the 2^−^ΔΔ^Ct^ method [23].

**Table 1:** Primer sequences for realtime-PCR

Name/Accession number	Forward primer	Reverse primer
Tracp-5b NM_001270889.1	5’CAGCTTCCACCCTGAGATTC3’	5’CGGTTCTGGCGATTTCTTTA3’
CathepsinK NM_031560.2	5’GACCAGCGAAGAAGTGGTTC3’	5’GACTCTGCCTTCCCACTCTG3’
Col1a1 NM_053304.1	5’AAGACCTCCCGCCTGCCCAT3’	5’CACGAAGCAGGCAGGGCCAA3’
Osteocalcin NM_013414.1	5’TGAGGACCCTCTCTCTGCTC3’	5’TGGACATGAAGGCTTTGTCA3’
β actin NM_031144.3	5′AGCCATGTACGTAGCCATCC3′	5′ACCTCATAGATGGGCACAG3′

### Histology

Hematoxylin and eosin (H&E) and tartrate-resistant acid phosphatase (TRAP) (Sigma-Aldrich, St Louis, MO) stainings were performed at day 28. The area of interest was delineated as 400μm^2^ area of interradicular bone of the first maxillary molar and located 200 μm from the mesiobuccal root of the first maxillary molar. For fluorescent microscopic imaging, the non-demineralized hemimaxillae at day 28 were processed in methyl methacrylate and cut using a grinder (Exakt Technologies, Oklahoma City, OK). The sections were evaluated using ImageJ program under a fluorescent microscope (EVOS FL, Life Technologies, Grand Island, NY).

### Serum biochemistry

On days 3, 7 and 28 of the study, the blood/serum from each animal was collected, processed and subject to osteoprotegerin (OPG) (Mybiosource, San Diego, CA) and receptor activator of nuclear factor kappa-B ligand (RANKL) (R&Dsystems, Minneapolis, MN) ELISA kits and the ratio was calculated.

### Statistical analysis

The experiments were performed in triplicate. Kruskal-Wallis analysis of variance was used to determine the mean difference among groups, and multiple comparisons were analyzed by Mann-Whitney-U tests with a significance level = 0.05.

## Results

### Distance of tooth movement

All rats were viable and showed no sign of lethargy after spring installation. Faxitron radiographs demonstrated a smaller distance between first and second molars in shockwave-treated rats (0.11 ± 0.07 mm) compared to the distance in the controls (0.44 ± 0.09 mm) (Fig. 1c, d) on day 28 of study. The μCT confirmed delayed tooth movement in shockwave-treated rats (Fig. 1e, f) but the significant difference between groups was found on day 28 (*P* < 0.05) (Fig. 1g).

### Gene expression study

**Fig. 2:**
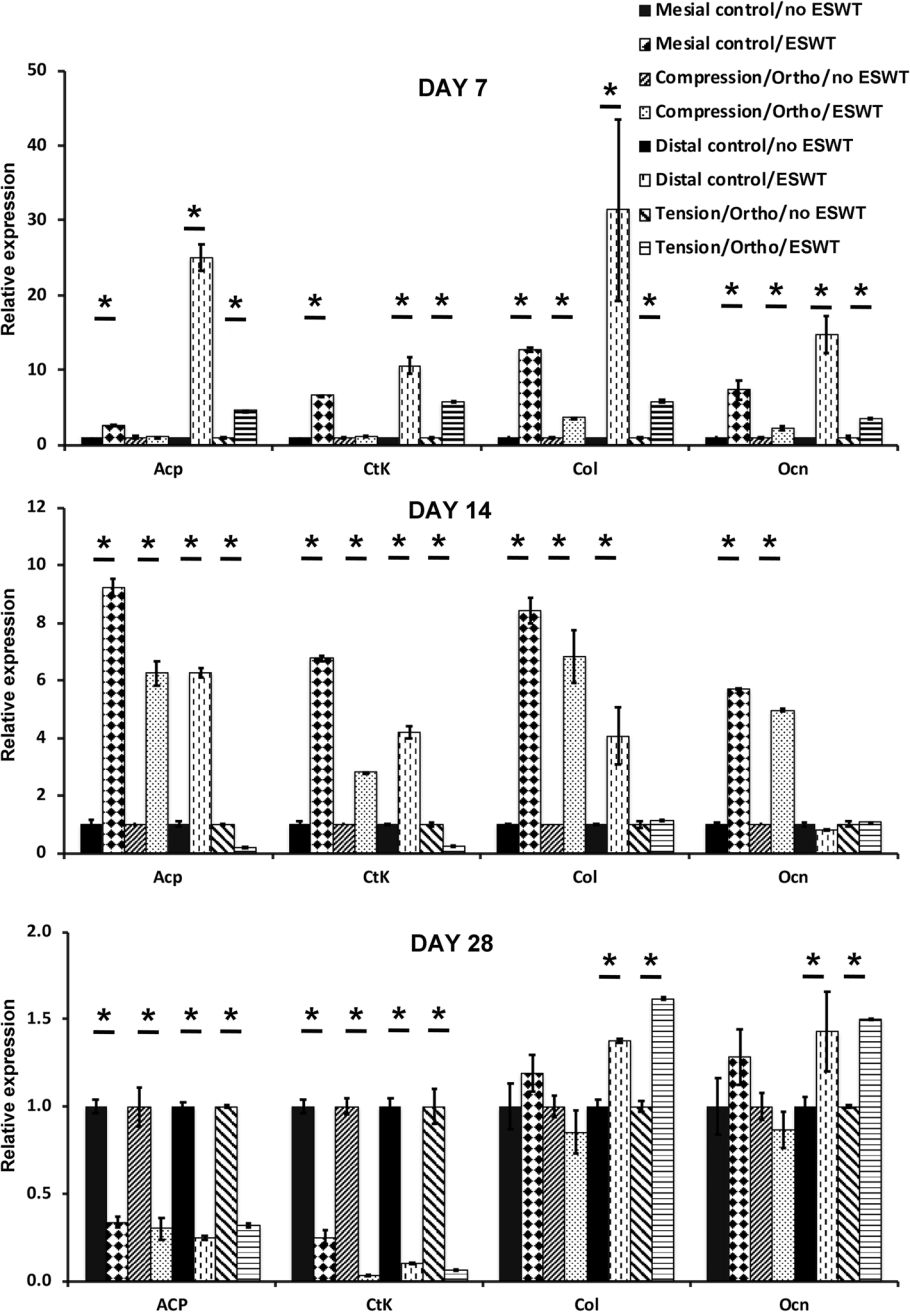
mRNA expression comparison of osteoclast markers and osteoblast markers of compression and tension alveolar bone samples from the non-shockwave control and shockwave-treated rats. (**a**) Day7, (**b**) Day14, (**c**) Day28. (ESWT: shockwave therapy, ortho: loading orthodontic force, Acp: acid phosphatase, Ctk: cathepsin K, Col: collagen I, Ocn: osteocalcin *:P < 0.05)

On day 7, increased expression of osteoclast markers: *cathepsinK (CtsK), acid phosphatase (Acp),* and osteoblast markers: *collagen I (Col1), osteocalcin (Ocn)* was observed in both compression and tension samples of the shockwave-treated rats, compared to the corresponding samples from the no ESWT (mesial and distal) controls (Fig. 2a). With the absence of orthodontic force, a 10–25-fold increase of all studied genes was observed on distal control sites of ESWT group compared to the corresponding site of no ESWT controls (*P* < 0.05) while a 5–10-fold increase was observed on mesial control sites of ESWT group compared to the no ESWT controls (*P* < 0.05). In the presence of orthodontic force, ESWT did not exhibit any positive effect on osteoclast markers and did exhibit only a 2–5-fold positive effect on osteoblast markers (*P* < 0.05). On day 14, with the absence of orthodontic force, a 5–10-fold increase expression of osteoclast and osteoblast markers was observed on the compression areas of ESWT groups compared to no ESWT groups (P < 0.05) (Fig. 2b). In the presence of orthodontic force, a 2–6-fold increase of osteoclast markers and osteoblast markers was observed on the compression side but a 3–8-fold decrease of osteoclast markers was observed on the tension side (*P* < 0.05). On day 28, the expression of all osteoclast markers decreased significantly in all groups. The positive effects of ESWT on osteoblast markers were observed on day 28 but a significant increase was found only in the tension sides of ESWT groups. These demonstrated that the positive effects of ESWT on osteoblasts lasted until the end of study (P < 0.05)(Fig. 2c) while ESWT exhibited negative effects on osteoclast at the end of study (*P* < 0.05) (Fig. 2c).

### Histological studies

**Fig. 3:**
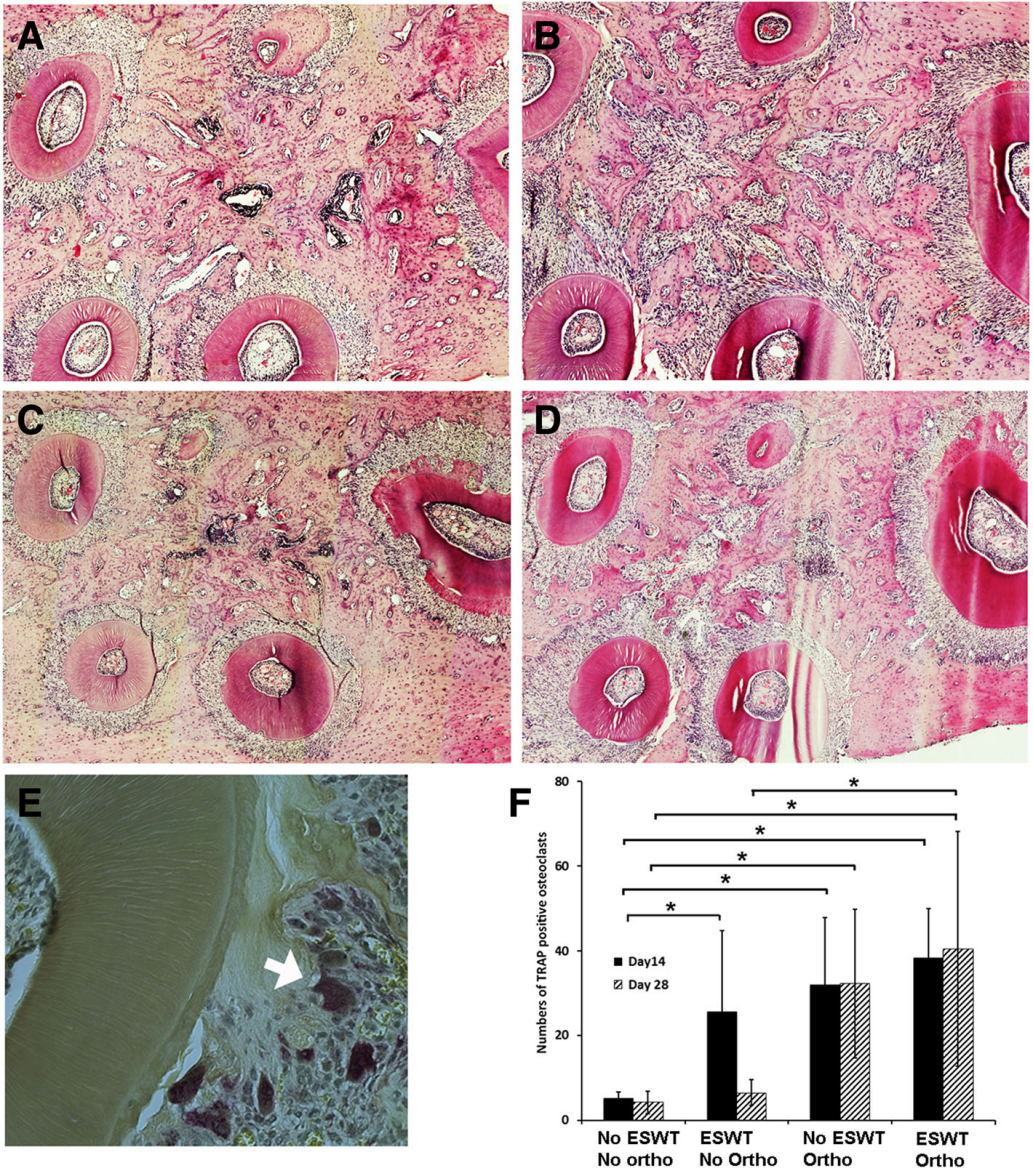
Histological sections of orthodontic-treated and/or shockwave-treated rats’ maxillae. **a**, **b**, **c**, **d** H&E sections of interradicular alveolar bone of a maxillary first molar from each group (**a**) no shockwave-treated and no orthodontic force control (no ESWT/no ortho), (**b**) no shockwave-treated with orthodontic force group (no ESWT/ortho), (**c**) shockwave-treated without orthodontic-treated group (ESWT/no ortho), (**d**) shockwave-treated with orthodontic-treated group (ESWT/ortho). **e** High magnification view of TRAP positive osteoclasts (arrow). **f** The number of osteoclasts of the studied areas of each studied group at day 14 and 28 (*:*P* < 0.05)

At day 28, in the presence of orthodontic force, both no ESWT (Fig. 3b) and ESWT (Fig. 3d) groups exhibited decreased periodontal ligament space width on the compression side but wide space on the tension side. Increased numbers of osteoclasts were observed at interradicular bone lacunae on the compression side and increased interradicular bone resorption was detected in both no ESWT and ESWT with orthodontic force (Fig. 3b, d) compared to those of no orthodontic force groups (Fig. 3a, c). Relative higher bone volume was detected in the ESWT group (Fig. 3c, d) compared to the no ESWT groups (Fig. 3a, b). TRAP staining (Fig. 3e) was performed to identify the osteoclasts at day 14 and 28. The result revealed significantly increased number of osteoclasts after a single treatment of shockwave treatment (ESWT/no ortho) when compared to the non-shockwave-treated and no orthodontic force control group (no ESWT/no ortho) (Fig. 3f). However, the increased number of osteoclasts was significant only on day 14**,** (Fig. 3f; *P* < 0.05) not on day 28 (Fig. 3f; *P* > 0.05). In the presence of orthodontic force, increased numbers of osteoclasts were detected in both no ESWT and ESWT groups (Fig. 3f; P < 0.05); however, there was no difference between both groups at any timepoint. In contrast, in the absence of orthodontic force, increased numbers of osteoclasts were detected only at day 14 of ESWT group (Fig. 3f; P > 0.05). The result may imply that the positive effects of ESWT on osteoclast numbers were only transient and synergistic in the presence of orthodontic force. However, in the presence of orthodontic force, the effect of ESWT on osteoclasts was not striking.

### Double bone labeling assay and serum biochemistry

**Fig. 4:**
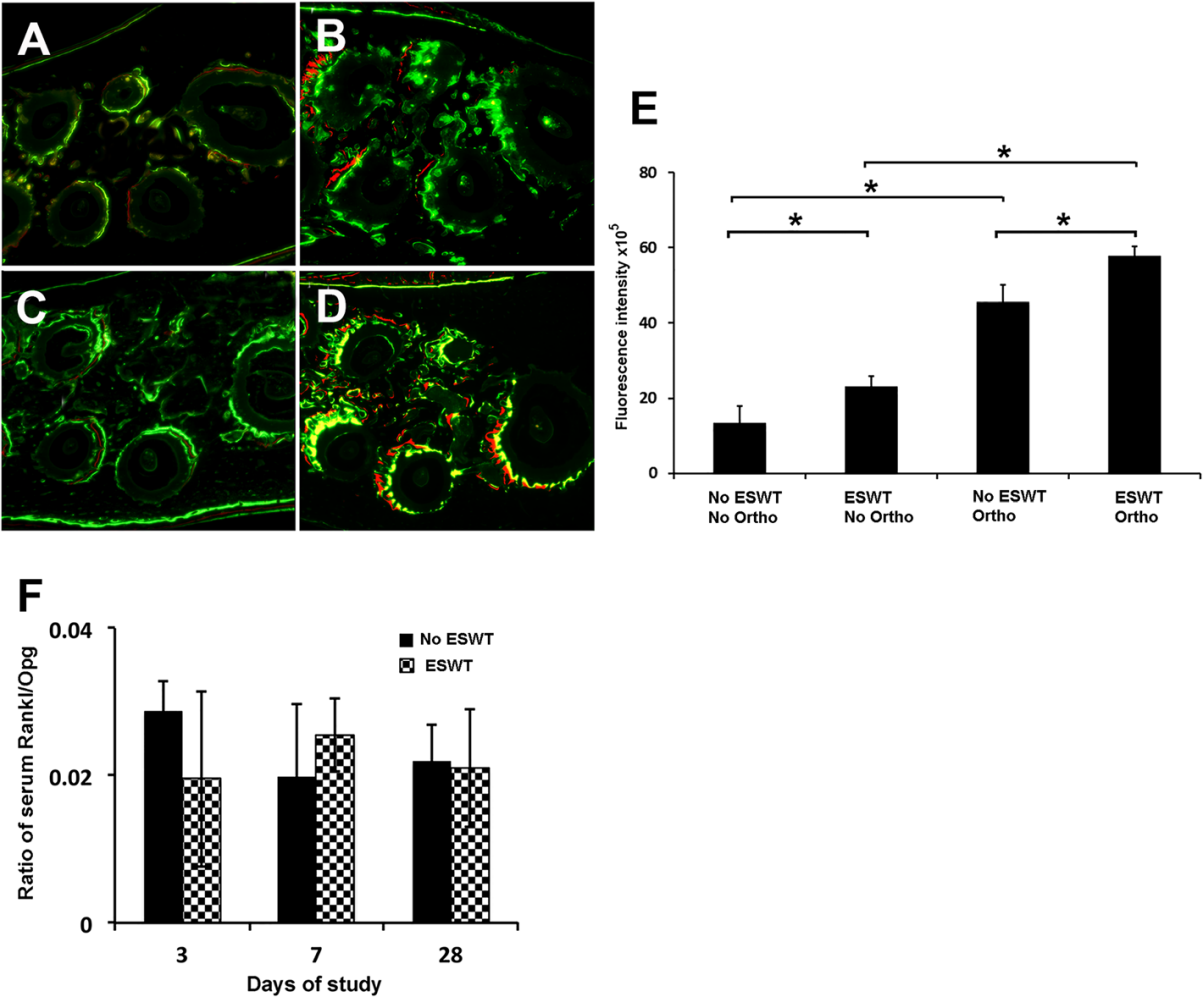
Double-fluorescence bone labeling with calcein (green color) and alizarin (red color) signal on cross sections of maxillae from 4 groups viewed under a fluorescent microscopy (**a**, **b**, **c**, **d**). **a** no shockwave treatment and no orthodontic treatment control (no ESWT/no ortho), (**b**) no shockwave treatment with orthodontic treatment group (no ESWT/ortho), (**c**) shockwave treatment without orthodontic treatment group (ESWT/no ortho), (**d**) shockwave treatment with orthodontic treatment (ESWT/ortho). Increased fluorescent signal was detected in shockwave-treated groups both orthodontic-treated (**c**) and non-orthodontic-treated groups **d**). **e** Comparison of fluorescence intensity of sections from each group. **f** The serum RANKL/OPG ratio of both shockwave- and non-shockwave groups at day 3, 7 and 28 of the study. No significant difference was found at any timepoints indicating no systemic effect after a single application of shockwave at early or late timepoint

At day 28, non-demineralized maxillae sections elucidated more prominent fluorescence signals from calcein and alizarin Red S in the ESWT groups (Fig. 4c, d) compared to the no ESWT groups (Fig. 4a, b), indicating increased bone apposition activity in the ESWT groups (Fig 4e). The orthodontic treatment alone increased bone remodeling activity compared to the non-orthodontic treatment control (Fig. 4a, e). ESWT alone (Fig. 4c, e) showed increased bone apposition as increased fluorescence signal compared to the non-orthodontic and non-shockwave control (Fig. 4a, e). The combination of shockwave treatment and orthodontic treatment showed the most prominent fluorescence signals among all samples (Fig. 4d, e). The ratio of serum RANKL and OPG from both ESWT and no ESWT rats was calculated at day 3, 7 and 28. No significant differences were found between both groups at any time-points indicating that the effect of shockwaves was localized and did not affect systemic activity of osteoclasts (Fig. 4f).

## Discussion

This study is the first to demonstrate that a single treatment of ESWT impedes tooth movement in rats, affects osteoclast differentiation and increases osteoblast activity. Increased levels of cytokines and osteoblast/clast markers play an important role in recruitment of osteoclasts and osteoblasts leading to active bone remodeling during tooth movement [24, 25]. Several previous studies showed that ESWT affects inflammatory and angiogenic cytokine expression and osteoblast activities with subsequent bone regeneration [26–30]. In this study, ESWT led to increased osteogenic activity and osteoblast marker expression similar to previous reports [19, 31]. The effects of ESWT were clearly observed when ESWT was used without orthodontic force. The positive effects of ESWT on osteoblasts lasted until the end of the study while the positive effects on osteoclast markers and numbers were transient. This phenomenon indicated imbalanced activities of osteoblast and osteoclast after ESWT treatment; however, the net positive effects of ESWT was on osteoblasts. In this study, though the focused applicator was used to limit the area of shockwave treatment, the effect on the surrounding tissues is difficult to eliminate. We evaluated the systemic effect of ESWT on other surrounding areas using ratio of serum OPG and RANKL to determine systemic activity of osteoclasts [32] during the study. No difference of the ratio of serum OPG and RANKL was found between ESWT and control groups indicating no systemic effect of ESWT after a single application. The effect**s** of ESWT application seemed to be local. Several studies reported that ESWT increased bone mineral content and bone mineral density in osteoporotic patients [31, 33]. In our study, H&E and double bone labeling demonstrated increased bone apposition and osteoblast markers expression of ESWT rats’ maxilla sections, which implicated the positive effects of ESWT on osteoblasts. Several studies demonstrated that ESWT promoted osteogenesis in human bone [34–36] and increased several growth factors for healing [36]. We speculate that the reduced tooth movement after a single treatment of ESWT was due to imbalanced activities of osteoblast**s** and osteoclast**s** during tooth movement.

### Limitations

Only one specific set of parameters of ESWT was used in the study. The effects of shockwave could be varied due to different parameters, including intensity and frequency of the shockwaves as well as the timing of application of shockwaves in relation to when tooth movement is started. The relapse animal model could be used to evaluate the effect of ESWT on relapse as well.

## Conclusions

1. A single application of ESWT of focused 500 impulses at EFD 0.1 mJ/mm^2^, with a pulse rate of 5 pulses per second applied at the time tooth movement was initiated impeded tooth movement in rats.
2. A single application of ESWT in this study promoted both osteoblastic and osteoclastic activities, however, there were net positive effects exhibited on osteoblasts along with imbalance of the bone remodeling process. ESWT had no systemic effect on osteoclast activity.

## Abbreviations

Acp: Acid phosphatase
Col1: Collagen I
CtsK: Cathepsin K
EFD: Energy flux density
ESWT: Extracorporeal shockwave therapy
H&E: Hematoxylin and eosin
IL-1β: Interleukin-1beta
Ocn: Osteocalcin
OPG: Osteoprotegerin
RANKL: Receptor activator of nuclear factor kappa-B ligand
RAP: Rapid acceleratory phenomenon
TRAP: Tartrate-resistant acid phosphatase
